# Supplementary material for: HIVprotI: an integrated web based platform for prediction and design of HIV proteins inhibitors
Source: J Cheminform. 2018 Mar 9;10:12. doi: 10.1186/s13321-018-0266-y (PMC5845081; doi:10.1186/s13321-018-0266-y)
Supplement: Supplementary file 2 — Additional file 2. Source code of HIVProtI web server. [file 13321_2018_266_MOESM2_ESM.zip › HIVprotI_Source-code/hivprotI/weka-3-6-6/doc/weka/classifiers/functions/SimpleLogistic.html]

SimpleLogistic


---


|  |  |  |
| --- | --- | --- |
| **Overview** \| **Package** \| **Class** \| **Tree** \| **Deprecated** \| **Index** \| **Help** \| **Weka's home** | |  |
| **PREV CLASS**   **NEXT CLASS** | **FRAMES**    **NO FRAMES**     **All Classes** |  |
| SUMMARY: NESTED \| FIELD \| CONSTR \| METHOD | DETAIL: FIELD \| CONSTR \| METHOD |  |


---


## weka.classifiers.functions Class SimpleLogistic

```
java.lang.Object
  weka.classifiers.Classifier
      weka.classifiers.functions.SimpleLogistic
```

**All Implemented Interfaces:**: java.io.Serializable, java.lang.Cloneable, AdditionalMeasureProducer, CapabilitiesHandler, OptionHandler, RevisionHandler, TechnicalInformationHandler, WeightedInstancesHandler

---

``` public class SimpleLogistic extends Classifier implements OptionHandler, AdditionalMeasureProducer, WeightedInstancesHandler, TechnicalInformationHandler ```

Classifier for building linear logistic regression models. LogitBoost with simple regression functions as base learners is used for fitting the logistic models. The optimal number of LogitBoost iterations to perform is cross-validated, which leads to automatic attribute selection. For more information see:
